# Fetuin-A Induced Suppression of PPAR Signaling: Molecular Insights and the Potential Regulatory Role of Fucosylation

**DOI:** 10.3390/cells15141262

**Published:** 2026-07-14

**Authors:** Yıldız Öner-İyidoğan, Hikmet Koçak

**Affiliations:** Department of Medical Biochemistry, Faculty of Medicine, Istinye University, 34408 Istanbul, Türkiye; hikmet.kocak@istinye.edu.tr

**Keywords:** Fetuin-A, PPARs, metabolic inflammation, fucosylation, NAFLD (redefined as MASLD)

## Abstract

**Highlights:**

**What are the main findings?**

- Fetuin-A may modulate PPAR signaling through multiple pathways, including TLR4–NF-κB, Wnt, ERK, and SIRT1/AMPK.
- The glycosylation status of fetuin-A, particularly fucosylation, may influence its role in metabolic inflammation.

**What are the implications of the main findings?**

- The Fetuin-A–PPAR interaction may provide a key mechanistic link between metabolic inflammation and progression of NAFLD to HCC.
- Fetuin-A and its glycoforms may represent potential biomarkers and targets for future research in metabolic disorders.

**Abstract:**

Metabolic diseases are characterized by a complex interplay between metabolic dysregulation and chronic low-grade inflammation. Fetuin-A (FetA), a liver-derived hepatokine, has emerged as a key mediator linking these processes through its pro-inflammatory and insulin resistance-promoting effects. Accumulating evidence indicates that FetA not only serves as a biomarker but also actively contributes to disease pathogenesis by modulating multiple signaling pathways. In this review, we present an overview of the molecular mechanisms underlying FetA-induced suppression of peroxisome proliferator-activated receptor (PPAR) signaling, a central regulator of metabolic homeostasis. Emerging evidence suggests that FetA may promote Toll-like receptor 4 (TLR4)-mediated inflammation, activate nuclear factor kappa B (NF-κB) signaling, suppress key energy regulators such as Sirtuin 1 (SIRT1) and AMP-activated protein kinase (AMPK), and inhibit PPAR activity through Wnt and extracellular signal-regulated kinase (ERK) pathways. These interconnected mechanisms may contribute to impaired lipid metabolism, increased insulin resistance, and metabolic inflammation. Furthermore, we highlight the role of FetA glycosylation, particularly fucosylation, as a regulatory layer influencing its biological activity. Fucosylated FetA may more effectively activate TLR4 signaling and suppress PPAR activity, suggesting functional heterogeneity among glycoforms. Overall, the FetA–PPAR interaction may represent a key mechanistic link between metabolic inflammation and disease progression.

## 1. Introduction

The metabolic and immune systems are two fundamental systems essential for maintaining human health and homeostasis. These regulatory pathways have evolved to operate in a highly coordinated and integrated manner. The coordinated interaction between these systems plays an important role in maintaining physiological homeostasis. However, disruption of this balance can lead to various pathological processes and contribute to the development of chronic metabolic diseases, including obesity, type 2 diabetes, and cardiovascular diseases [1].

According to the classical definition, inflammation is an acute response to tissue injury characterized by prominent clinical signs such as swelling, redness, pain, and increased temperature. However, the inflammatory features observed in metabolic diseases differ substantially from this classical definition [2]. Inflammation associated with obesity and related metabolic disorders is typically low-grade, chronic, and systemic, and does not conform to the conventional definition of inflammation [1]. Therefore, in recent years, this distinct inflammatory state induced by caloric excess and metabolic stress has been termed “metabolic inflammation” or “metaflammation.” Although this process shares common signaling mechanisms with classical inflammatory pathways, it exhibits distinct pathophysiological characteristics in terms of its initiating factors and biological outcomes [1].

Metabolic diseases, particularly insulin resistance (IR), non-alcoholic fatty liver disease (NAFLD, recently redefined as metabolic dysfunction-associated steatotic liver disease, MASLD), and their advanced stages such as steatohepatitis and hepatocellular carcinoma (HCC), represent a serious threat to public health and impose a substantial burden on healthcare systems worldwide. In the pathogenesis of these conditions, in addition to classical metabolic disturbances, chronic low-grade inflammation has been recognized as a major contributing factor [1].

Proteins secreted from the liver, known as hepatokines, are defined as molecules that exert significant effects on systemic metabolic regulation [3]. Fetuin-A (α2-Heremans–Schmid glycoprotein) defined as a hepatokine, has attracted considerable attention due to its strong association with IR and lipid metabolism. Fetuin-A (FetA) has been reported to stimulate the production of inflammatory cytokines from adipocytes and macrophages, suggesting its potential role as a biomarker for the identification and monitoring of chronic inflammatory diseases [4,5,6]. Stefan et al. reported that increased serum FetA levels in humans are significantly associated with reduced insulin sensitivity and increased hepatic fat accumulation [7]. In the same study, FetA levels decreased following weight loss, further supporting its active role in metabolic regulation [7].

FetA has been shown to contribute to Toll-like receptor 4 (TLR4)-mediated inflammatory signaling suggesting that it functions not only as a biomarker but also as an active participant in metabolic inflammation [8].

On the other hand, peroxisome proliferator-activated receptors (PPARs) are ligand-activated transcription factors that belong to the nuclear receptor superfamily [9]. This term was first introduced in the early 1990s based on their ability to bind compounds that induce peroxisome proliferation [10]. In mammals, three principal PPAR isoforms have been identified: PPARα (NR1C1), PPARβ/δ (NR1C2), and PPARγ (NR1C3). These receptors form heterodimers with the retinoid X receptor (RXR) and bind to PPAR response elements (PPREs), thereby regulating the expression of genes involved in adipogenesis, lipid metabolism, inflammation, and metabolic homeostasis [11]. Notably, PPARγ has emerged as an important regulator of adipogenesis and insulin sensitivity, while also exerting significant anti-inflammatory effects [12].

Previous studies indicate that FetA and PPAR signaling pathways have largely been investigated independently, and studies examining their molecular interplay within an integrated framework remain limited.

Recent experimental studies have demonstrated that FetA may suppress PPARγ activity through multiple signaling pathways. Activation of the Wingless/Integrated (Wnt) signaling pathway by FetA, leading to reduced PPARγ expression [13], increased phosphorylation of PPARγ via the Rat sarcoma (Ras)–mitogen-activated protein kinase kinase (MEK)–extracellular signal-regulated kinase (ERK) signaling pathway [14], and inhibition of major energy sensors such as Sirtuin 1 (SIRT1) and AMP-activated protein kinase (AMPK) [15] collectively suggest that the relationship between FetA and PPARγ is multidimensional.

Taken together, these findings suggest that FetA may suppress PPAR signaling—particularly PPARγ—through inflammatory and metabolic pathways, thereby exerting its metabolic effects via this mechanism. This review aims to systematically examine how FetA modulates PPAR signaling through inflammatory and metabolic pathways and to discuss the potential pathological consequences of the FetA–PPAR interaction.

## 2. PPAR Signaling in Metabolic and Inflammatory Regulation

PPARs, as members of the nuclear receptor superfamily, play a central role in regulating the expression of numerous target genes involved in metabolic processes [16]. These target genes are involved in the regulation of lipid metabolism, glucose homeostasis, and inflammatory processes [9,11]. The tissue-specific expression patterns and diverse functional properties of PPAR isoforms establish them as principal regulators of metabolic homeostasis [11,17].

PPAR-α: PPAR-α is primarily expressed in the liver, heart, skeletal muscle, and brown adipose tissue, and serves as a major regulator of fatty acid β-oxidation and lipid catabolism [9,11,17]. PPAR-α activation reduces lipotoxicity via increased fatty acid oxidation and protects against NAFLD by limiting hepatic lipid accumulation [18].

PPAR-α also suppresses inflammation by inhibiting pro-inflammatory gene expression [19]. Accordingly, PPAR-α is a major regulator of metabolic inflammation.

PPAR-γ: PPAR-γ is predominantly expressed in adipose tissue and serves as a central regulator of adipogenesis, lipid storage, and insulin sensitivity [9,20,21]. PPAR-γ has been shown to regulate adiponectin expression in adipose tissue and to modulate insulin sensitivity [22,23]. Furthermore, PPAR-γ exerts potent anti-inflammatory effects by promoting macrophage polarization toward the M2 phenotype, thereby contributing to the suppression of chronic inflammation [12]. Owing to these properties, PPAR-γ is regarded as a promising therapeutic target for the management of both metabolic disorders and inflammatory conditions.

PPAR-δ: PPAR-δ is ubiquitously expressed in multiple tissues and serves as an important regulator of energy expenditure, fatty acid oxidation, and cellular energy homeostasis [11,17]. Activation of PPAR-δ has been demonstrated to modulate metabolic processes, notably through the enhancement of fatty acid oxidation in skeletal muscle [11,24]. Furthermore, PPAR-δ has been shown to modulate inflammatory responses and to promote cellular adaptation to metabolic stress via macrophage activation [25].

PPARs are not only metabolic regulators but also transcriptional modulators that actively participate in the control of inflammation. In particular, PPAR-γ has been shown to suppress the activity of the pro-inflammatory transcription factor nuclear factor kappa B (NF-κB), thereby reducing the expression of inflammatory genes [26]. However, increased activation of inflammatory signaling pathways has also been reported to suppress PPAR expression and activity [19]. This bidirectional interaction plays a decisive role in the regulation and maintenance of metabolic inflammation.

Given their central roles in metabolic and inflammatory processes, PPARs are highly susceptible to upstream regulatory signals. In particular, factors influencing inflammation and energy metabolism may suppress PPAR activity, thereby contributing to the development of metabolic diseases [9,11,17,27,28,29]. Accordingly, accumulating evidence suggests that FetA, as a hepatokine, may play a regulatory role in modulating PPAR activity through multiple signaling pathways.

## 3. Fetuin-A: A Key Hepatokine in Metabolic Inflammation

FetA is a ~63 kDa glycoprotein predominantly synthesized in the liver and secreted into the circulation. It has been increasingly recognized as a hepatokine with a significant role in the pathogenesis of metabolic diseases [3,6,8,30]. Although initially identified as an calcification inhibitor, FetA is now widely defined as an active regulator of energy metabolism and inflammation. Recent studies have shown that its synthesis is primarily regulated in the liver and influenced by metabolic and inflammatory conditions, including IR, lipid accumulation, and cytokine signaling [7,8,31,32,33,34].

Clinical studies have consistently suggested a strong association between elevated serum FetA levels and IR [8,30]. Notably, measurements performed using the euglycemic–hyperinsulinemic clamp method have demonstrated that elevated FetA levels correlate with reduced insulin sensitivity [7].

Previous studies have also demonstrated a positive correlation between FetA levels and hepatic fat accumulation [6,7,35,36]. These findings support a significant role for FetA in the pathogenesis of NAFLD. Additionally, the decrease in FetA levels following weight loss suggests that this molecule is dynamically regulated in relation to metabolic status [7].

The metabolic effects of FetA are largely mediated through inflammatory signaling pathways. Pal et al. has suggested that FetA promotes TLR4-dependent inflammatory signaling, leading to NF-κB activation and increased production of pro-inflammatory cytokines [8]. These findings suggest that FetA is not merely a biomarker but also an active mediator of inflammation. Consistent with this, our 2026 study revealed that the FetA/adiponectin ratio was significantly associated with adipose tissue inflammation in overweight individuals, along with adipokines such as progranulin and C1q/TNF-related protein-3 [37]. In addition, findings from our previous study indicate that serum FetA levels are associated with inflammatory status and arginine metabolism in obesity [38]. The inflammatory effects of FetA extend beyond the systemic level to the cellular level. Studies in pancreatic β-cells have demonstrated that FetA, in conjunction with palmitate, induces cellular damage and apoptosis through the activation of the TLR4–c-Jun N-terminal kinase (JNK)–NF-κB signaling pathway [39]. This process contributes to impaired insulin secretion by inducing β-cell apoptosis through JNK activation and NF-κB-mediated inflammatory signaling [39]. Taken together, these observations may highlight a strong link between FetA and metabolic inflammation.

The metabolic effects of FetA involve basic cellular regulators of energy homeostasis. FetA has been demonstrated to upregulate tumor necrosis factor alpha (TNF-α), which in turn promotes SIRT1 degradation and leads to a reduction in AMPK activity [15]. These alterations have been demonstrated to disrupt energy homeostasis and contribute to reduced insulin sensitivity [15].

When considered collectively, the effects of FetA on inflammation, IR and energy metabolism highlight its significant role in the regulation of metabolic inflammation. FetA may promote inflammation via TLR4, suppress major energy sensors such as SIRT1 and AMPK, increase cellular damage, and exacerbate IR. These multifaceted effects position FetA as a potential upstream regulator in the pathogenesis of metabolic diseases.

The broad impact of FetA on inflammatory and metabolic pathways suggests that it does not act in isolation but rather interacts with other common regulatory systems. Hence, current findings indicate that FetA may suppress PPAR activity through multiple signaling mechanisms, thereby amplifying metabolic inflammation.

## 4. Molecular Mechanisms of Fetuin-A-Induced Suppression of PPAR Signaling

Recent studies have demonstrated that, beyond acting as an inflammatory mediator, FetA can suppress PPAR signaling -one of the key regulators of metabolic homeostasis- through multiple pathways. Although these mechanisms may appear distinct, they ultimately converge on a common regulatory outcome, as summarized below.

TLR4–NF-κB Interaction and Suppression of PPAR Signaling: In a study by Pal et al., FetA was demonstrated to interact with TLR4 through complex formation with free fatty acids, thereby activating inflammatory signaling pathways [8]. Activation of TLR4 has been shown to stimulate the NF-κB signaling pathway, resulting in increased secretion of TNF-α and other pro-inflammatory cytokines [8]. These findings provide a mechanistic basis for the indirect regulation of PPAR signaling by FetA. In inflammatory conditions, both the expression and activity of PPARγ are markedly suppressed, and this inhibition constitutes a significant mechanism driving the persistence of metabolic inflammation [12]. Therefore, FetA may be considered a potential upstream regulator that indirectly suppresses PPAR activity through the pro-inflammatory microenvironment generated via TLR4 signaling.

Suppression of PPARγ via the Wnt Signaling Pathway: The effects of FetA on PPARγ are not confined to inflammatory mechanisms. Supporting this, Agarwal et al. reported that FetA activates the Wnt3a signaling pathway, thereby suppressing PPARγ expression and leading to a reduction in adiponectin levels [13]. These findings suggest that FetA exerts its metabolic effects through an alternative mechanism involving the direct targeting of PPARγ.

Ras–MEK–ERK Pathway and PPARγ Phosphorylation: One of the most prominent effects of FetA on PPARγ occurs at the post-translational level. In a study by Das et al., FetA was shown to stimulate TNF-α synthesis, thereby activating the Ras–MEK–ERK signaling pathway and inducing phosphorylation of PPARγ at the Ser273 residue [14]. This modification has been reported to reduce the transcriptional activity of PPARγ, decrease adiponectin expression, and promote IR [14]. This mechanism may inform that FetA suppresses PPARγ at both the expression and functional levels.

Indirect Suppression of PPAR via Energy Sensors: The metabolic effects of FetA involve essential regulators of cellular energy homeostasis, particularly SIRT1 and AMPK. FetA has been demonstrated to downregulate SIRT1 protein levels through TNF-α, thereby leading to suppression of AMPK activity [15]. These alterations lead to reduced activity of peroxisome proliferator-activated receptor gamma coactivator 1-alpha (PGC-1α), impaired mitochondrial function, and attenuation of PPARγ signaling. Collectively, these findings may indicate that FetA indirectly modulates PPAR activity through its effects on energy metabolism.

The effects of FetA are not confined to PPARγ. In caloric restriction models, reduced FetA levels have been shown to correlate with increased PPAR-α activity [40]. This relationship promotes increased fatty acid oxidation and attenuated inflammation, indicating that FetA serves as a regulator of hepatic metabolism [40].

Integration of Mechanisms: FetA–PPAR Interaction: Taken together, these findings suggest that FetA suppresses PPAR signaling through multiple, interconnected mechanisms:

- TLR4–NF-κB: inflammatory signaling,
- Wnt signaling: transcriptional repression,
- ERK activation: post-translational modification,
- Reduced SIRT1/AMPK activity: indirect regulation via energy sensors.

**Figure 1:**
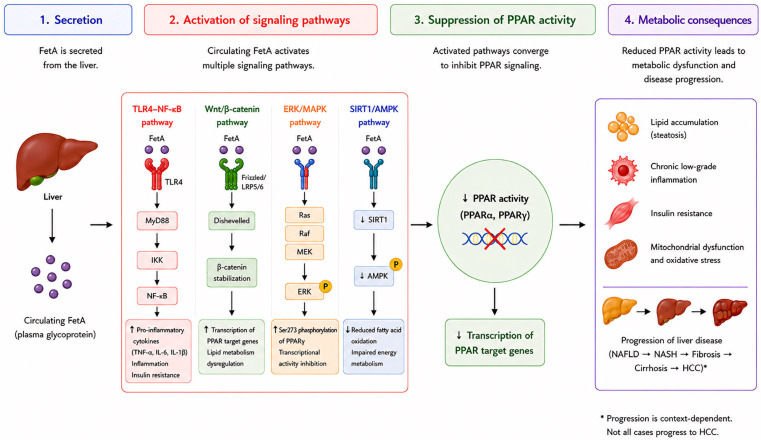
Hypothetical model of Fetuin-A-mediated modulation of PPAR signaling and metabolic disease progression. The figure illustrates a hypothetical model in which circulating FetA activates multiple signaling pathways that converge to suppress PPAR activity. Reduced PPAR signaling may contribute to metabolic dysfunction, including lipid accumulation, chronic inflammation, insulin resistance, and mitochondrial impairment, and may promote progression from NAFLD to NASH, fibrosis, cirrhosis, and HCC. **Abbreviations:** AMPK, AMP-activated protein kinase; ERK, Extracellular signal-regulated kinase; FetA, Fetuin-A; HCC, Hepatocellular carcinoma; IKK, IκB kinase; IL-1β, Interleukin-1 beta; IL-6, Interleukin-6; LRP5/6, LDL receptor-related protein 5/6; MAPK, Mitogen-activated protein kinase; MEK, Mitogen-activated protein kinase kinase; MyD88, Myeloid differentiation primary response 88; NAFLD, Non-alcoholic fatty liver disease; NASH, Non-alcoholic steatohepatitis; NF-κB, Nuclear factor kappa B; PPAR, Peroxisome proliferator-activated receptor; PPARα, Peroxisome proliferator-activated receptor alpha; PPARγ, Peroxisome proliferator-activated receptor gamma; Raf, Rapidly accelerated fibrosarcoma kinase; Ras, Rat sarcoma virus protein; SIRT1, Sirtuin 1; TLR4, Toll-like receptor 4; TNF-α, Tumor necrosis factor-alpha; Wnt, Wingless/Integrated signaling pathway.

Collectively, these pathways may converge to reduce PPAR activity and promote metabolic dysfunction. In this framework, FetA can be regarded as an upstream regulator integrating multiple signaling networks. By operating at the interface of inflammatory and metabolic pathways, FetA can act as a central modulator of PPAR activity through diverse mechanisms. A schematic summary is presented in Figure 1.

## 5. Fetuin-A Fucosylation/Glycosylation: A Potential Regulator of PPAR Signaling

FetA is a plasma glycoprotein with a highly complex glycosylation profile, and its functional properties are determined not only by its polypeptide backbone but also by the glycan moieties it carries [41]. FetA is characterized by two N-linked glycosylation sites (Asn138 and Asn158) and multiple O-linked glycan structures, and alterations in these glycans may profoundly influence its biological interactions and functional properties [42]. Protein glycosylation is profoundly altered in cancer and chronic inflammatory diseases, and terminal modifications such as fucosylation and sialylation have been increasingly recognized as potential biomarkers in various malignancies [43,44,45,46,47]. Elevated fucosylation levels of serum proteins have been consistently reported in liver diseases and hepatocellular carcinoma, and these alterations have been shown to carry significant diagnostic value [48]. In support of the clinical relevance of altered FetA glycosylation, Betesh et al. identified fucosylated FetA as a potential biomarker for cholangiocarcinoma, further suggesting that disease-associated changes in FetA glycoforms may have diagnostic utility beyond hepatocellular carcinoma [49].

Protein glycosylation is a common post-translational modification that regulates ligand–receptor interactions, cell adhesion, and signal transduction, particularly through terminal glycan modifications. Because FetA is a plasma glycoprotein with complex N- and O-linked glycan structures, the functional properties of FetA are closely dependent on its glycosylation status. In particular, terminal modifications such as fucosylation can modulate receptor function and immune signaling pathways, including Toll-like receptor complexes, by altering signaling strength [50,51,52]. The glycosylation profile of FetA is influenced by genetic polymorphisms and disease states, with elevated fucosylation levels particularly observed under inflammatory conditions [41]. These findings suggest that the biological effects of FetA glycoforms may be heterogeneous and that their functional outcomes could depend on their specific glycosylation status.

Accordingly, it can be proposed that distinct glycoforms of FetA differentially regulate its interaction with TLR4 and its capacity to activate inflammatory signaling pathways. However, studies directly investigating the receptor-level effects of specific glycosylation variants of FetA remain limited, and this relationship has yet to be systematically elucidated. To provide a structured overview of the available findings, the main mechanisms linking FetA to PPAR signaling are summarized in Table 1.

**Figure 2:**
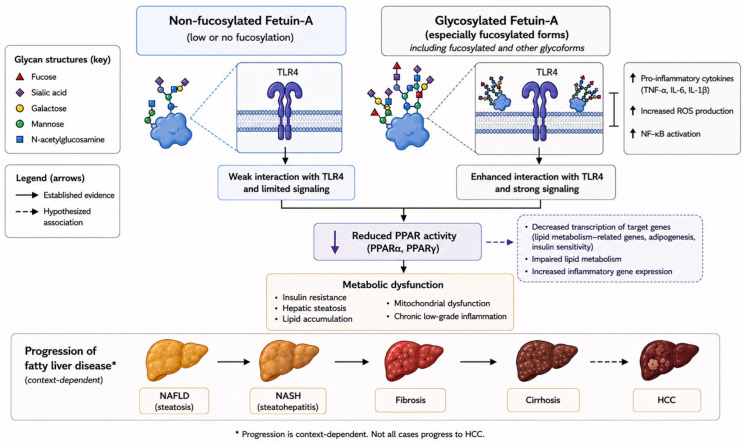
Hypothetical model of Fetuin-A glycoforms in modulating TLR4-mediated inflammatory signaling and PPAR activity. Non-fucosylated FetA shows weak interaction with TLR4 and limited inflammatory signaling, whereas glycosylated FetA, particularly fucosylated forms, enhances TLR4 activation, promoting NF-κB signaling, pro-inflammatory cytokine production, and ROS generation. These effects are associated with reduced PPAR activity, contributing to impaired lipid metabolism and insulin sensitivity. Consequently, metabolic dysfunction develops, including insulin resistance, lipid accumulation, mitochondrial dysfunction, and chronic inflammation. In liver disease, these alterations may promote progression from NAFLD to NASH, fibrosis, cirrhosis, and HCC. **Abbreviations:** HCC, Hepatocellular carcinoma; IL-1β, Interleukin-1 beta; IL-6, Interleukin-6; NAFLD, Non-alcoholic fatty liver disease; NASH, Non-alcoholic steatohepatitis; NF-κB, Nuclear factor kappa B; PPAR, Peroxisome proliferator-activated receptor; PPARα, Peroxisome proliferator-activated receptor alpha; PPARγ, Peroxisome proliferator-activated receptor gamma; ROS, Reactive oxygen species; TLR4, Toll-like receptor 4; TNF-α, Tumor necrosis factor-alpha.

Current studies suggest that both the total levels of FetA and its glycosylation status may serve as important determinants of its biological activity. In particular, fucosylated FetA may potentially enhance TLR4 activation, amplify inflammatory responses, and more effectively suppress PPAR signaling [8,48]. This hypothesis supports a proposed model in which FetA exerts heterogeneous effects on PPAR, with distinct glycoforms driving differential biological outcomes. Future studies using purified glycoforms, defucosylated FetA, TLR4 reporter assays, TLR4 blockade, and PPAR transcriptional activity assays are needed to validate this proposed model. These approaches would help clarify whether the observed effects are directly mediated by specific glycoforms or reflect broader changes in glycosylation patterns. A schematic overview of this hypothesis is presented in Figure 2.

## 6. From Metabolic Inflammation to NAFLD and Hepatocellular Carcinoma

The progression of metabolic disorders may, in specific contexts such as NAFLD, follow a multistep process characterized by lipid accumulation, chronic inflammation, and, in some cases, malignant transformation. This process is especially governed by the interplay between metabolic and inflammatory signaling pathways.

NAFLD, characterized by triglyceride accumulation in hepatocytes and closely associated with IR, serves as a representative model of this paradigm. However, lipid accumulation alone does not fully account for disease progression, as inflammatory processes play a central role in its pathogenesis [53,54]. Based on current concepts, NAFLD is conceptualized within the framework of the “multiple parallel hits” model, in which adipose tissue-derived signals, hepatokines, and inflammatory mediators collectively contribute to disease progression [55].

Consequently, FetA emerges as an important hepatokine contributing to NAFLD pathogenesis by promoting systemic inflammation and IR [8,56,57].

PPARs, particularly PPAR-α and PPAR-γ, may play a central role in hepatic lipid metabolism. PPAR-α activation enhances fatty acid oxidation, whereas PPAR-γ regulates lipid storage and insulin sensitivity [9,11,17,58]. FetA-mediated suppression of PPAR activity through multiple mechanisms may accelerate NAFLD progression by attenuating fatty acid oxidation, promoting lipid accumulation, and disrupting metabolic homeostasis [57,59].

Non-alcoholic steatohepatitis (NASH, recently redefined as metabolic dysfunction-associated steatohepatitis, MASH), the progressive stage of NAFLD, is characterized by hepatocellular injury, inflammation, and fibrosis, and is associated with pronounced activation of inflammatory signaling pathways [55]. In particular, TLR4-mediated signaling has been demonstrated to play a central role in the pathogenesis of hepatic inflammation and fibrosis [60]. Persistent FetA-mediated activation of this pathway may further contribute to disease progression by sustaining chronic inflammatory responses [8,60]. Moreover, the detrimental effects of FetA on IR and energy metabolism suggest that this molecule contributes to the persistence of inflammation within the hepatic microenvironment [7]. This process may ultimately contribute to hepatocellular injury and the activation of fibrogenic pathways.

Chronic inflammation and metabolic dysfunction may be major determinants in HCC development. The majority of HCC cases arise in the context of chronic liver disease, with NASH emerging as one of the fastest-growing etiological contributors, particularly in association with metabolic syndrome [61]. These findings can underscore the central role of metabolic inflammation in tumor biology. Accordingly, chronic inflammation, oxidative stress, and dysregulated proliferative signaling may establish a microenvironment conducive to tumor development [62].

The combined pro-inflammatory effects of FetA and its inhibitory impact on PPAR signaling suggest that this molecule may play a role in establishing a tumor-promoting microenvironment. Given the anti-inflammatory and anti-proliferative properties of PPAR-γ, suppression of this pathway may enhance cellular proliferation, sustain inflammatory signaling, and drive tumor progression. Hence, the FetA–PPAR interaction may represent an important mechanistic link between metabolic inflammation and malignant transformation. It is therefore plausible that FetA-mediated inflammation and PPAR suppression contribute to the progression from NAFLD to NASH and ultimately to HCC.

## 7. Translational and Therapeutic Implications

The FetA–PPAR interaction not only advances our understanding of disease pathogenesis but also provides novel opportunities for the diagnosis and treatment of metabolic diseases. A better understanding of FetA–PPAR interactions may provide new insights for the development of targeted interventions in metabolic diseases. Clinical studies have consistently demonstrated that serum FetA levels are associated with IR, hepatic fat accumulation, and metabolic disorders [5,8,59]. These findings suggest that FetA may serve as a potential biomarker for early diagnosis and risk stratification in metabolic diseases.

During the progression of liver diseases, particularly HCC, profound alterations in serum glycoprotein glycosylation patterns occur, with increased fucosylation representing a hallmark feature. Elevated levels of both core and outer-arm fucosylation have been strongly associated with HCC and demonstrated significant diagnostic value [48,63,64,65,66]. These findings suggest that fucosylated glycoproteins are not only indicative of disease-associated alterations but also may represent potential biomarkers. Moreover, the glycosylation profile of FetA is influenced by genetic polymorphisms and disease states, with elevated fucosylation levels particularly observed under inflammatory conditions [41]. These observations suggest that the biological effects of FetA glycoforms may be heterogeneous and highly dependent on their specific structural modifications. Accordingly, fucosylated FetA may represent a novel biomarker candidate with enhanced diagnostic specificity compared to total FetA. However, further investigation is required to elucidate the functional significance of these glycoforms, particularly in disease pathogenesis.

Lectin-based analytical approaches designed to assess protein glycosylation patterns provide a powerful framework for biomarker discovery. By integrating antibody-based capture with lectin-mediated detection, these methods enable the characterization of glycan modifications independently of total protein abundance [67]. Applying this approach to FetA may enable the quantitative assessment of fucosylated FetA, potentially offering a valuable tool for early diagnosis and monitoring of disease progression in metabolic disorders.

Therefore, PPAR agonists, particularly PPAR-γ agonists such as thiazolidinediones, are widely used in the treatment of metabolic diseases due to their insulin-sensitizing and anti-inflammatory effects [12,68], through increasing adiponectin levels, activation of PPAR-γ contributes to the maintenance of metabolic homeostasis and suppression of inflammatory responses [69].

However, considering the pro-inflammatory and IR-promoting properties of FetA [7,8], it may be proposed that FetA indirectly suppresses PPAR-mediated metabolic responses. FetA-targeted interventions may represent a potential future strategy to improve PPAR-based therapies.

Elucidating the FetA–PPAR interaction may facilitate the development of innovative combination therapeutic strategies. Hence, the clinical use of PPAR activators, anti-inflammatory agents, and FetA-targeted interventions may enhance therapeutic efficacy, particularly in complex metabolic disorders such as NAFLD and NASH.

The feasibility of measuring circulating FetA levels supports its utility as a non-invasive biomarker. Assessment of FetA and its glycoforms in serum and potentially other biological fluids, including saliva, may broaden its clinical applicability. Furthermore, the integration of biomarker data with clinical and demographic variables may enable the development of artificial intelligence-based predictive models.

Taken together, the FetA–PPAR interaction constitutes a promising translational axis with significant implications for disease pathogenesis, biomarker discovery, and targeted therapeutic development. However, these implications remain speculative and require further experimental and clinical validation.

## 8. Conclusions and Future Perspectives

Understanding the molecular mechanisms driving metabolic disease pathogenesis may be important for the development of novel diagnostic and therapeutic strategies. Accordingly, the hypothesis presented in this review indicates that FetA may function not only as a hepatokine or biomarker but also as an essential integrative regulator of inflammatory and metabolic signaling networks.

Current findings suggest that FetA may activate TLR4-mediated inflammation, suppress SIRT1/AMPK pathways, and inhibit PPAR activity through Wnt and ERK signaling pathways. Collectively, these mechanisms may converge on the suppression of PPAR signaling, resulting in disruption of metabolic homeostasis. Therefore, the “FetA–PPAR interaction” proposed in this review provides an integrative conceptual framework for understanding metabolic inflammation by linking inflammation, energy metabolism, and lipid regulation, and by helping to reconcile fragmented findings in the literature.

Notably, the biological effects of FetA may be determined not only by its circulating levels but also by its glycosylation status. In particular, the hypothesis that fucosylated FetA exerts distinct regulatory effects on inflammatory signaling and PPAR activity represents a fundamental direction for future research. Further studies are warranted to characterize the functional properties of different FetA glycoforms, validate their effects on PPAR signaling at molecular and cellular levels, and assess the diagnostic and prognostic value of fucosylated FetA in clinical settings. Integration of systems-level approaches may further elucidate this complex regulatory network.

From a therapeutic perspective, targeting FetA-mediated suppression of PPAR signaling may offer novel opportunities for the treatment of metabolic diseases. Combination strategies incorporating PPAR agonists and FetA-targeted interventions may be particularly beneficial in complex disorders such as NAFLD and related conditions.

In conclusion, the FetA–PPAR interaction represents a novel regulatory mechanism at the core of metabolic inflammation, with significant implications for both mechanistic insight and translational applications.

## Figures and Tables

**Table 1:** Summary of experimental studies investigating the relationship between Fetuin-A and PPAR signaling pathways.

Mechanism	Model	Findings	PPAR Isoform	Strength of Evidence	References
TLR4–NF-κB activation	In vitro (adipocytes, macrophages)	FetA–FFA complexes activate TLR4 signaling and increase pro-inflammatory cytokine production	PPARγ	Strong	[8]
Wnt/β-catenin pathway	Cell culture	FetA-mediated Wnt activation is associated with reduced PPARγ expression	PPARγ	Moderate	[13]
ERK (Ras–MEK–ERK pathway)	Cell culture	FetA induces phosphorylation of PPARγ, leading to reduced transcriptional activity	PPARγ	Moderate	[14]
SIRT1/AMPK pathway	Cell culture	FetA reduces SIRT1 and AMPK activity, impairing energy metabolism	Indirect	Moderate	[15]
Glycosylation (fucosylation)	Clinical biomarker study and indirect glycoprotein evidence	Disease-associated fucosylated FetA has been identified as a potential biomarker, while fucosylation may modulate receptor interactions and inflammatory signaling	Potential	Limited/Hypothetical	[48,49,50]

**Abbreviations:** FetA, Fetuin-A; PPAR, peroxisome proliferator-activated receptor; TLR4, Toll-like receptor 4; NF-κB, nuclear factor kappa B; FFA, free fatty acids; ERK, extracellular signal-regulated kinase; Wnt, Wingless/Integrated signaling pathway; SIRT1, sirtuin 1; AMPK, AMP-activated protein kinase.

## Data Availability

No new data were created or analyzed in this study.
